# Role of Hematopoietic Stem Cell Transplantation in Acute Promyelocytic Leukemia

**DOI:** 10.3389/fonc.2021.614215

**Published:** 2021-03-18

**Authors:** Jaime Sanz, Pau Montesinos, Miguel A. Sanz

**Affiliations:** ^1^ Department of Hematology, Hospital Universitari i Politècnic La Fe, Valencia, Spain; ^2^ Centro de Investigación Biomédica en Red de Cáncer, Instituto Carlos III, Madrid, Spain

**Keywords:** acute promyelocytic leukaemia, hematopoietic (stem) cell transplantation (HCT), all-trans retinoic acid (ATRA), arsenic trioxide, relapse

## Abstract

The indication of hematopoietic stem cell transplantation (HSCT) in acute promyelocytic leukemia (APL) has evolved historically from a widespread use in front-line therapy during the pre-ATRA era to a virtual rejection of this indication for patients treated with modern treatments. HSCT in first complete remission could only be considered for an extremely small fraction of patients with persistent MRD at the end of consolidation or for those who relapse. In the pre-ATO era, relapsed patients were usually treated with readministration of ATRA and chemotherapy as salvage therapy, generally containing high-dose cytarabine and an anthracycline, followed by further post-remission chemotherapy and/or HSCT. ATO-based regimens are presently regarded as the first option for relapsed APL. The selection of the most appropriate post-remission treatment option for patients in second CR (CR2), as well as the modality of HSCT when indicated, depends on several variables, such as pre-transplant molecular status, duration of first remission, age, and donor availability. Although with a moderate level of evidence, based on recent retrospective studies, autologous HSCT would be at present the preferred option for consolidation for patients in molecular CR2. Allogeneic HSCT could be considered in patients with a very early relapse or those beyond CR2. Nevertheless, the superiority of HSCT as consolidation over other alternatives without transplantation has recently been questioned in some studies, which justify a prospective controlled study to resolve this still controversial issue.

## Introduction

Modern treatment approaches for patients with newly diagnosed APL, using the combination of all-trans retinoic acid (ATRA) with either arsenic trioxide (ATO), chemotherapy or both, result in 90% to 95% complete remission (CR) rates with virtual absence of primary resistance, and 85% to 90% rates of long-term survival (1). Different to most subtypes of acute myeloid leukemia, these outstanding results in APL have been obtained without consolidating patients in first CR (CR1) with hematopoietic stem cell transplantation (HSCT). In this context, HSCT has virtually ceased to play any role in CR1 and relegated to consolidate patients in CR2 or beyond after salvage therapy for relapsed APL (1–3).

In this review article, we will discuss the clinical outcomes of HSCT in patients with APL and discuss the issues related to this indication, including the preferred choice of donor, source of hematopoietic stem cells, and conditioning regimen, among other controversial matter. We will also address the main prognostic factors for transplant outcomes that have been identified. We aim to provide insight into decision making regarding the optimal use of HSCT in patients with APL.

## HSCT has No Role in Front-Line Therapy for Newly Diagnosed APL

The indication of HSCT in patients with APL has evolved historically from a widespread use of this procedure in front-line therapy during the pre-ATRA era to a virtual rejection of this indication when patients are treated with modern treatments containing ATRA. Except for the beginning of the ATRA era, in which many groups still continued to indicate an HSCT in CR1 (4, 5), this has gradually been abandoned and explicitly rejected by the European LeukemiaNet (ELN) recommendations (1, 2) and the National Comprehensive Cancer Network (NCCN) guidelines (3).

Currently, the general consensus is that HSCT has no role patients in CR1, even in high-risk patients.

## HSCT as Consolidation Therapy is Better than Non-HSCT in Relapsed APL

Only a few small retrospective and uncontrolled studies have compared the therapeutic efficacy of HSCT versus consolidation without transplantation in relapsed patients with APL (**Table 1**). Most of these studies showed that transplanted patients had a higher event-free survival (EFS), ranging from 61% to 83% for auto-HSCT (6, 7, 9) and from 41% to 71% for allo-HSCT (6, 9, 10), compared to those not transplanted, ranging from 30% to 45% (6, 7, 10). It should be noted, however, that these differences were statistically significant in only two of these studies (6, 7).

**Table 1:** Hematopoietic stem cell transplantation (autologous and/or allogeneic) compared with non-transplantation in relapsed APL.

Reference	Study period	Salvage therapy	No. of patients			Event-free survival	Overall survival	Relapse rate	Time
			Auto	Allo	Non-HSCT				
De Botton et al. (6)	1992-2001	ATRA+CHT	50	23	49	61 vs 52 vs 30 P = 0.002	60 vs 52 vs 40 P = 0.005	87 vs 92 vs 38^a^ P = 0.005	7-year
Thirugnanam et al. (7)	1998-2006	ATO-based	14	–	19	83 vs 34 P = 0.001	100 vs 39 P = 0.001	7 vs 63^b^ P < 0.0001	5-year
Lengfelder et al. (8)	2003-2011	ATO-based	60	33	55	NA	77 vs 79 vs 59 P = 0.09	37 vs 39 vs 59 P = 0.05	3-year
Pemmaraju et al. (9)	1980-2010	Miscellaneous	10	17	16	69 vs 41 vs NA P = 0.45	86 vs 49 vs 40 P = 0.48	NA	7-year
Fujita et al. (10)	1997-2002	ATRA+CHT	6	21	30	42 vs 71 vs 45 NS	83 vs 76 vs 75 NS	58 vs 10 vs 51^c^ P = 0.007	7-year
Ganzel et al. (11)	<2000-2011	ATO-based	140	–	67	NA	78 vs 42 P < 0.001	NA	5-year

NA, not available; NS, not significant.

a Relapse-free survival.

b Crude relapse rate.

c Allo vs auto: P = 0.007; allo vs non-HSCT: P = 0.009.

The superiority of transplantation versus non-transplantation was even more apparent for overall survival (OS), which ranged from 60% to 100% for auto-HSCT (6–11) and 49% to 79% for allo-HSCT (6, 8–10), while it was 39% to 75% for those not transplanted (6–11). Except from two small studies (9, 10), the remaining ones showed a significantly higher OS (6–8, 11). Not without reason, it can be argued that differences in survival outcomes could be explained, at least in part, by a selection bias, since transplantation is ruled out in a sizable proportion of patients because they are considered clinically unfit. However, this explanation would not be enough, since practically all the studies have shown a higher relapse rate in patients undergoing consolidation treatment without transplantation (6, 7, 10), including a retrospective ELN study in relapsed APL treated with ATO-based regimens (8). It should be noted, however, that some groups have recently reported prolonged remissions in series of patients relapsing after ATRA plus chemotherapy treated with ATO plus ATRA without transplant.

Based on the previously mentioned studies, the ELN recommendations (1) and the NCCN guidelines (3) consider transplantation as the best option to consolidate patients in CR2 or beyond after salvage therapy for relapsed APL. This is, however, an unresolved issue, since some recent reports question the need for transplantation, at least in patients who achieve molecular remission with ATO and ATRA (1) or ATO, ATRA, mitoxantrone and bortezomib (12). Therefore, a prospective controlled study is warranted to address this still controversial issue.

## The Choice of Autologous or Allogeneic HSCT

Once it has been shown that consolidation with HSCT, either auto-HSCT or allo-HSCT, results in better survival outcomes than non-transplant strategies for patients in CR2 or beyond, the next question that arises is the appropriate type of transplant to be done. The choice of autologous or allogeneic transplantation should be based on the modality that has shown higher efficacy in this setting. Again a few small, retrospective and uncontrolled studies have compared the therapeutic efficacy of auto-HSCT and allo-HSCT in relapsed patients with APL (**Table 2**). None of these studies was able to demonstrate superiority of one modality of HSCT over the other in EFS, except from an analysis of the Acute Leukemia Working Party (ALWP) of the European Blood and Marrow Transplantation (EBMT) that has been recently published (16). In this large study that included 341 and 228 APL patients in CR2 who underwent auto-HSCT and allo-HSCT, respectively, EFS was significantly higher in the former group (75% vs 55%). Auto-HSCT was also superior to allo-HSCT in terms of OS, not only in this study, but also in other large studies (6, 15). The most likely explanations for finding a clearer benefit in OS than in EFS could be, on the one hand, because the potential benefit of a graft-versus-leukemia (GVL) effect in patients undergoing allo-HSCT would be widely counterbalanced by a higher transplant related mortality (TRM) systematically reported in that setting compared with patients undergoing auto-HSCT (5, 6, 9, 10, 13–15). In fact, no study was able to demonstrate a significant reduction in the relapse rate, while the two largest studies reported a significantly higher rate of TRM in the allo-HSCT (15). On the other hand, a second relapse after auto-HSCT is probably a clinical situation with a higher chance of subsequent salvage as compared to a second relapse after allo-HSCT. This would also explain that, in most series, the increase in the OS rate with respect to the EFS is significantly greater in patients undergoing auto-HSCT compared to those undergoing allo-HSCT (9, 10, 15).

**Table 2:** Autologous versus allogeneic hematopoietic stem cell transplantation in relapsed APL.

Reference	Study period	Salvage therapy	No. of patients		Event-free survival	Overall survival	Relapse rate	TRM rate	Time
			Auto	Allo					
Sanz et al. (5)	1993-2003	ATRA+CHT	195	137	51 vs 59^a^	NA	37 vs 17 P = NA	16 vs 24 P = NA	5-year
De Botton et al. (6)	1992-2001	ATRA+CHT	50	23	61 vs 52 P = 0.11	60 vs 52 P = 0.04	79 vs 92^a^ P = 0.19	6 vs 39 P = NA	7-year
Kohno et al. (13)	1999-2004	Miscellaneous	15	13	69 vs 46^b^ P = 0.4	76 vs 46 P = 0.2	21 vs 9 P = NS	20 vs 46 P = NA	4-year
Lengfelder et al. (8)	2003-2011	ATO-based	60	33	NA	77 vs 79 NS	37 vs 39 NS	NA	3-year
Pemmaraju et al. (9)	1980-2010	Miscellaneous	10	17	69 vs 41 P = 0.45	86 vs 49 P = NS	30 vs 18^c^ P = NA	20 vs 47 P = NA	7-year
Fujita et al. (10)	1997-2002	ATRA+CHT	6	21	42 vs 71 NS	83 vs 76 NS	58 vs 10^b^ P = 0.007	0 vs 19	7-year
Alimoghaddam et al. (14)	1989-2011	ATO-based	11	29	52 vs 62^b^ P = 0.64	47 vs 66 NS	NA	0 vs 21^c^	5-year
Holter Chakrabarty et al. (15)	1995-2006		62	232	63 vs 50^b^ P = 0.1	75 vs 54 P = 0.002	30 vs 18 P = 0.4	7 vs 31 P < 0.001	5-year
Sanz et al. (16)	2004-2018		341	228	75 vs 55 P = 0.001	82 vs 64 P = 0.001	23 vs 28 P = 0.28	3 vs 17 P = 0.001	2-year

NA, not available; NS, not significant.

a Leukemia-free survival.

b Disease-free survival.

c Crude relapse rate.

In addition to comparative studies providing evidence that auto-HSCT is a better treatment strategy than allo-HSCT in relapsed APL (**Table 2**), other large case series have also demonstrated the outstanding efficacy and feasibility of that procedure after salvage therapy with ATO-based treatment (17), improving the outcomes of those with ATRA plus chemotherapy-based approaches (18).

## The Choice of Stem Cell Source and Conditioning Regimen

As far as we know, there are no prospective and controlled studies comparing results between peripheral blood and bone marrow as a source of hematopoietic progenitors in the specific context of APL. However, it is a fact that peripheral blood has been by far the preferred stem cell source used in most studies analyzing auto-HSCT in this disease (**Table 3**). Except for the previous EBMT study conducted between 1993 and 2003 (5), in which the proportion of patients transplanted with peripheral blood was 53%, all the remaining comparative studies reported percentages in the narrow range between 86% and 100%, including the last EBMT study in which the proportion increased to 92% (16). It should be noted, however, that the few retrospective studies that compared bone marrow with peripheral blood showed that the stem cell source did not affect either of the outcomes (5, 21). In contrast, the most common stem cell source in the allo-HSCT setting was bone marrow (range, 64% to 87%), except for a couple of series that reported a lower proportion of 47% and 18% (9). Interestingly, most studies in patients with APL undergoing allo-HSCT include a very high proportion of those from matched sibling donor (MSD), sometimes up to 100%, which is significantly higher than would be due to the average availability of this type of donor (roughly 30%). In some studies, this reflects the eligibility criteria established for the study, which exclude transplants from alternative sources and donors, but in other studies it may reflect the attitude of many reluctant physicians to choose alternative donors when a MSD is not available. Probably also, the retrospective studies currently available in relapsed APL do not reflect the great advances and growing use of alternative donors that are taking place in the field of allo-HSCT. It is a fact that significant differences in survival in the past between MSD, matched unrelated mismatched related and unrelated donors are gradually being curtailed.

**Table 3:** Stem cell source and conditioning intensity.

Reference	Auto-HSC					Allo-HSCT						
	No. of Patients	Source, %		Conditioning, %		No. of Patients	Source, %		Conditioning, %		Donor	
		BM	PB	MAC	RIC		BM	PB	MAC	RIC	MSD	Non-MSD
Sanz et al. (5) {Sanz:2007jv}	195	47	53			137	64	36			100	0
De Botton et al. (6)	50	14	86			23	87	13			78	22
Kohno et al. (13)	15	3	12	15	0	13	10	3	12	1	7	6
Thirugnanam et al. (7)	14	0	100	100	0	–	–	–				
Ferrara et al. (19)	13	0	100	100	0							
Lengfelder et al. (8)	60	NA	NA*	100	0	33	NA	NA	80	20	NA	NA
Pemmaraju et al. (9)	10	25	75	100	0	17	47	53	100		71	29
Fujita et al. (10)	6	0	100	100	0	21	71	19	90	10	38	62
Alimoghaddam et al. (14)	11	20	80	NA	NA	29			NA	NA	100	0
Ramadan et al. (20)	–	–	–	–	–	31	42	58	97	3	58	42
Holter Chakrabarty et al. (15)	62	12	88	89	8	232	66	34	92	7	53	47
Ganzel et al. (11)	140	NA	NA	NA	NA	–	–	–	–	–	–	–
Sanz et al. (16)	341	5	95	86	14	228	18	79	68	32	57	43
Yanada et al. (17)	35	0	100	100	0	–	–	–	–	–	–	–
Yanada et al. (18)	184	4	96	100	0	–	–	–	–	–	–	–
Yanada et al. (21)	443	4	96			–	–	–				

*PB in almost all cases.

NA, not available.

Regarding conditioning intensity, myeloablative conditioning regimens (MAC) were almost universally used for auto-HSCT, except a few patients who received reduced intensity regimen (RIC) (15). Although MAC was also the most common preparative regimen in allo-HSCT, RIC is increasingly used for older and medically unfit patients up to 32% (16). Unfortunately, data following RIC in APL are currently lacking.

As shown in **Table 4**, TBI-based regimens were preferred for auto-HSCT in the CIBMTR registry data (76%) (15) and in a single center study (10). In contrast, non-TBI-based regimens were preferred in most recent report of the EBMT registry (85%) (16), the Japan Adult Leukemia Study Group (100%) (17), and the Japanese Society for Hematopoietic Cell Transplantation (JSHCT) (96%) (21), as well as other single center studies (7, 13, 19). None of these studies with an appropriate sample size have addressed the comparison of results according to the conditioning regimen to draw significant conclusions.

**Table 4:** Conditioning regimens.

Reference	Auto-HSC						Allo-HSCT						
	TBI, %	Chemotherapy, %					TBI, %	Chemotherapy, %					
		BU/CY	BU/FLU	BU/MEL	FLU/MEL	Other		BU/CY	BU/FLU	BU/MEL	FLU/MEL	Other	
Sanz et al. (5)	29						53						
De Botton et al. (6)	56	34	0	8	2	0	74	24	0	0	0	0	
Ferrara et al. (19)	0	31	0	0	0	69							
Kohno et al. (13)	27	40	0	0	0	33	85	0	0	0	0	15	
Thirugnanam et al. (7)	0	100	0	0	0								
Pemmaraju et al. (9)	0	50	20	0	0	30	18	18	24	–	18	24	–
Fujita et al. (10)	100	–	–	–	–	–	68	32	–	–	–	–	
Alimoghaddam et al. (14)	–	–	–	–	–	100	–	90	10	–	–	–	–
Ramadan et al. (20)	–	–	–	–	–	–	50	50					
Holter Chakrabarty et al. (15)	76						50						
Sanz et al. (16) {Sanz:2020ve}	15	46	4	13	0	22	34	28	18	1	6	13	
Yanada et al. (17)	0			100									
Yanada et al. (18)	0	8		76		16							
Yanada et al. (21)	4	17		55		25*							

*BU/ETP/Ara-C (16%).

In allo-HSCT, TBI and non-TBI-based regimens were equally distributed in the CIBMTR registry data (15) and the previous EBMT study (5). However, TBI-based regimens were preferred in in some studies (6, 10, 13) and non-TBI in others (9), including the most recent of the EBMT registry (16).

## The Role of Transplantation in Patients with Minimal Residual Disease Positive After Salvage Therapy

It is generally accepted that patients undergoing transplantation with minimal residual disease (MRD) positive have a worse prognosis than in MRD-negative status. The clinical situation in which APL patients do not achieve MRD-negative status can occur in two different scenarios, one in which after first-line consolidation treatment the patient does not achieve molecular remission (primary molecular resistance or molecular persistence) and another in which the patient does not achieve molecular remission after salvage consolidation therapy (secondary molecular resistance). For the extremely low fraction of patients in the former scenario, given their poor prognosis, unless they are promptly managed aggressively prior to the occurrence of a hematologic relapse (22), allo-HSCT has traditionally been considered as the first option when patients are suitable for transplantation. It should be noted that molecular persistence at this point was already uncommon in early studies (3%-4%) (23), but has almost disappeared (<1%) in patients receiving state-of-the art treatments with either ATRA plus ATO (24) or ATRA plus chemotherapy-based approaches (25). As far as we know, the only study whose objective was to analyze the outcomes in patients with molecular persistence at the end of first-line consolidations was reported in 2004 and with a very small sample size (22). Four patients undergoing allo-HSCT were alive in hematologic and molecular remission at 64, 92, 98 and 118 months, while three patients treated with chemotherapy followed by auto-HSCT were alive in hematologic and molecular remission at 64, 96 and 98 months. These three patients were in molecular remission at the time of auto-HSCT.

Regarding the second scenario, that is, in patients MRD-positive who underwent transplantation during CR2, data are even scarcer. Some studies in the past showed data that today could provide clues for the interpretation of more current, apparently paradoxical data. While Meloni et al. reported 7 patients with relapsed APL undergoing auto-HSCT being MRD-positive and all patients relapsed within 9 months of transplantation (26), two single case reports showed long-term molecular remission in patients transplanted in CR2 after receiving MRD-positive autografts (27, 28). It was subsequently described in four patients who had molecular evidence of disease in at least one of the harvested samples and remained MRD negative after auto-HSCT (29). This could be explained, among other hypothesis, by the non-clonogenic nature of the PML/RARA-positive cells present in the graft. In fact, the persistence of differentiating PML/RARA-positive cells and spontaneously cleared during follow-up is a common event in patients receiving ATRA. In addition, long-term hematopoiesis after autologous HSCT would be sustained by the subset of CD34+/CD38− progenitor cells administered, and these immature progenitors have been shown to lack the PML/RAR rearrangement in APL patients.

Some recent studies have analyzed transplant outcomes according to MRD status before transplantation. Despite the bias inherent in the generally recommended strategy of limiting autologous transplantation for MRD-negative patients, relegating allogeneic transplantation for MRD-positive patients, these studies have provided some interesting data. Surprisingly, as in the CIBMTR study (15), the most recent EBMT study also found that transplant outcomes in MRD-positive recipients were not statistically different between autologous and allogeneic HSCT (16). Although these data should be interpreted with caution for a number of reasons, such as sample size and selection bias, among others, both studies have confirmed that a proportion of MRD-positive recipients can achieve long-term disease control not only undergoing allo-HSCT, but surprisingly also after auto-HSCT. This interesting finding apparently contradicts the notion that performing auto-HSCT in patients MRD positive is hopelessly doomed to failure. A recent study of the JSHCT, in a large series of APL patients undergoing auto-HSCT, with 35 being MRD-positive and 293 MRD-negative pre-transplantation, reported no association between MRD status and TRM, relapse, and OS rates (21). The cumulative incidence of relapse at 5 years was 10.3% (95% CI 7.1–14.3%) for patients with negative PML-RARA, and 8.9% (95% CI 2.3–21.3%) for patients with positive PML-RARA at transplant. In contrast to the study by Meloni et al. (26), in which bone marrow was the stem cell source, the vast majority of the patients included in the IBMTR, EBMT and JSHCT studies used peripheral blood stem cells (15, 21). These findings raise the question of the feasibility of performing auto-HSCT in patients MRD-positive when using peripheral blood as a of stem cell source and deserve to be confirmed in prospective studies.

## Prognostic Factors

A few registry-based studies have addressed a reliable analysis of prognostic factors associated to transplant outcomes in APL. Although originally introduced for allo-HSCT in chronic myeloid leukemia (30), and later demonstrated its applicability to acute myeloid leukemia and other malignant and non-malignant diseases (22), the classical EBMT risk score has never been tested for patients with APL. However, some of the few available studies on prognostic factors in APL have demonstrated the predictive value of some of the factors considered in the EBMT risk score. Thus, age (> 40 years) and a shorter duration of CR1 adversely influenced overall mortality in the CIBMTR study (15, 21), while in the EBMT study (16), age assessed as a continuous variable (per 10 years) and also a shorter interval from diagnosis to transplant showed an adverse impact not only on TRM (only age), but also in relapse risk, leukemia-free and overall survival. In another retrospective pan-European study in patients receiving ATO as salvage therapy (8), in addition to molecular persistence, a shorter duration of CR1 (<18 months) had an adverse impact on transplant outcomes (LFS and OS) in multivariable analysis.

The stage of the disease as such, another factor considered in the EBMT risk score, has been little studied in APL. In fact, as far as we know, only a retrospective study carried out in five Italian transplant centers has addressed this issue in a relatively small series (20). The study reported the outcome of 31 APL patients who underwent allo-HSCT in CR2 (n=15) or beyond (n=16), with OS being worse in patients with more advanced disease, but at the limit of statistical significance (p = 0.05) in the univariate analysis, while relapse rate was not statistically significant. Although it is expected that other factors considered in the EBMT risk score, such as donor type and donor recipient sex combinations, could also be predictive of transplant outcomes in APL, we are not aware of any study that has analyzed these factors in this disease.

Regarding prognostic factors in auto-HSCT, in addition to the duration of CR1, it has recently been suggested that salvage therapy with ATO is associated with a delayed hematopoietic recovery after transplantation. This association was initially suggested in two cases (31), but later confirmed in a retrospective review of 58 APL patients undergoing auto-HSCT at 21 institutions in the United States and Japan (32). This study found that ATO exposure prior to hematopoietic progenitor cell collection has negative impact on hematopoietic recovery after auto-HSCT. Fortunately, this delay in hematopoietic recovery does not appear to have a significant impact on TRM and other transplant outcomes.

Patients with CNS or other extramedullary relapse have classically been associated with a poorer outcome than those with isolated bone marrow relapse (33). However, recent data have not been able to demonstrate a negative impact of extramedullary disease on transplant outcomes after salvage therapy (8, 11).

## Conclusions

The high cure rate currently obtained in patients with APL using modern treatments with ATRA plus chemotherapy or ATRA plus ATO point out that there is no role for HSCT in front-line therapy. The indication of HSCT has been relegated as consolidation of relapsed patients who achieve second CR after salvage therapy whenever possible. Although with a moderate level of evidence, based on recent retrospective studies, autologous HSCT would be the preferred option for consolidation for patients in molecular CR2. Allogeneic HSCT could be considered in patients with a very early relapse or those beyond CR2. Nevertheless, the superiority of HSCT as consolidation over other alternatives without transplantation has recently been questioned in some studies, which justify a prospective controlled study to resolve this still controversial issue.

## Author Contributions

All authors have contributed equally. All authors contributed to the article and approved the submitted version.

## Conflict of Interest

The authors declare that the research was conducted in the absence of any commercial or financial relationships that could be construed as a potential conflict of interest.
